# High and low frequency stimulation of the subthalamic nucleus induce prolonged changes in subthalamic and globus pallidus neurons

**DOI:** 10.3389/fnsys.2013.00073

**Published:** 2013-12-18

**Authors:** Hagar Lavian, Hana Ben-Porat, Alon Korngreen

**Affiliations:** ^1^The Leslie and Susan Gonda Interdisciplinary Brain Research Center, Bar-Ilan UniversityRamat Gan, Israel; ^2^The Mina and Everard Goodman Faculty of Life Sciences, Bar-Ilan UniversityRamat Gan, Israel

**Keywords:** subthalamic nucleus, globus pallidus, high frequency stimulation, low frequency stimulation, basal ganglia

## Abstract

High frequency stimulation (HFS) of the subthalamic nucleus (STN) is widely used to treat the symptoms of Parkinson’s disease (PD) but the mechanism of this therapy is unclear. Using a rat brain slice preparation maintaining the connectivity between the STN and one of its target nuclei, the globus pallidus (GP), we investigated the effects of high and low frequency stimulation (LFS) (HFS 100 Hz, LFS 10 Hz) on activity of single neurons in the STN and GP. Both HFS and LFS caused changes in firing frequency and pattern of subthalamic and pallidal neurons. These changes were of synaptic origin, as they were abolished by glutamate and GABA antagonists. Both HFS and LFS also induced a long-lasting reduction in firing frequency in STN neurons possibly contending a direct causal link between HFS and the outcome DBS. In the GP both HFS and LFS induced either a long-lasting depression, or less frequently, a long-lasting excitation. Thus, in addition to the intrinsic activation of the stimulated neurons, long-lasting stimulation of the STN may trigger prolonged biochemical processes.

## Introduction

High frequency stimulation (HFS) of the subthalamic nucleus (STN) is widely used to treat various basal ganglia disorders, particularly Parkinson’s disease (PD; Limousin et al., 1995; Starr et al., 1998). Because both STN lesions and HFS lead to similar amelioration of PD symptoms (Bergman et al., 1990; Benazzouz et al., 1993), it is commonly thought that the alleviating effect of HFS stems from partial or complete inhibition of the STN neurons. Supporting this hypothesis, some *in vivo* studies have shown that HFS in the STN suppressed the firing rate of STN neurons (Tai et al., 2003; Filali et al., 2004). Similarly, HFS of the STN in brain slice preparations inhibited subthalamic neurons (Beurrier et al., 2001; Magarinos-Ascone et al., 2002), supporting the view of inhibition of the STN as the underlying cause for alleviation of PD symptoms. Note, however, that such inhibitory effects have not yet been demonstrated to be specific to stimulation at therapeutic parameters. Furthermore, other *in vivo* studies found that HFS in the STN led to increased glutamate concentration (Windels et al., 2000) or elevated firing rate in STN target nuclei, indicating increased subthalamic activity (Hashimoto et al., 2003). Finally, using optogenetics to selectively inhibit subthalamic neurons did not affect motor symptoms in hemi-parkinsonian rats (Gradinaru et al., 2009). Thus, the effects of HFS in the STN on the firing frequency of its neurons and other basal ganglia neurons are unresolved.

HFS may alleviate PD by changing the firing pattern of STN neurons rather than their firing rate, as HFS in an irregular pattern is not effective in treating PD, as oppose to regular stimulation (Dorval et al., 2010). Indeed, *in vivo* HFS of the STN causes changes in the timing of the firing of STN neurons (Meissner et al., 2005) and of neurons in the external and internal globus pallidus (GP; Hashimoto et al., 2003; Moran et al., 2011).

In contrast with the alleviating effect of HFS, low frequency stimulation (LFS) may worsen PD symptoms (Timmermann et al., 2004). Can this be correlated to cellular changes in STN neurons as observed for HFS? Therefore, to investigate the effects of STN stimulation, we compared the effects of HFS and LFS on the STN and on one of its target nuclei, the GP. Pallidal neurons not only receive glutamatergic input from the STN (Smith and Parent, 1988), but also innervate STN neurons via GABAergic synapses (Shink et al., 1996). We use these two reciprocally connected nuclei as a system for understanding the dynamic changes occurring during repetitive stimulation. Whole-cell recordings were made simultaneously from pallidal and subthalamic neurons in a rat brain slice preparation preserving the connectivity between these two nuclei. Both HFS and LFS led to similar prolonged depression of subthalamic firing. A similar long-term depression was also seen in pallidal neurons but also, less frequently, a long–term excitation.

## Materials and methods

### *In vitro* slice preparation

Brain slices were obtained from 17–22 days old Wistar rats as previously described (Stuart et al., 1993; Bugaysen et al., 2010). Rats were killed by rapid decapitation according to the guidelines of the Bar-Ilan University animal welfare committee. This procedure was approved by the national committee for experiments on laboratory animals at the Israeli Ministry of Health. The brain was quickly removed and placed in ice-cold artificial cerebrospinal fluid (ACSF) containing (in mM): 125 NaCl, 4 KCl, 25 NaHCO_3_, 1.25 Na_2_HPO_4_, 2 CaCl_2_, 2 MgCl_2_, 25 glucose, and 0.5 Na-ascorbate (pH 7.4 with 95% O_2_/5% CO_2_). Thick sagittal slices (370 μm) were cut on an HR2 Slicer (Sigman Electronic, Germany) and transferred to a submersion-type chamber, where they were maintained for the remainder of the day in ACSF at room temperature. Experiments were carried out at 37°C, the recording chamber was constantly perfused with oxygenated ACSF.

### *In vitro* electrophysiology

Individual GP and STN neurons were visualized using infrared differential interference contrast (IR-DIC) microscopy. Whole-cell recordings were obtained from the soma of GP and STN neurons using patch pipettes (4–8 MΩ) pulled from thick-walled borosilicate glass capillaries (2.0 mm outer diameter, 0.5 mm wall thickness, Hilgenberg, Malsfeld, Germany). The standard pipette solution contained (in mM): 140 K-gluconate, 10 NaCl, 10 HEPES, 4 MgATP, 0.05 SPERMIN, 5 l-glutathione, 0.2 EGTA, and 0.4 GTP (Sigma, pH 7.2 with KOH). The reference electrode was an Ag–AgCl pellet placed in the bath. Voltage signals were amplified by an Axopatch-200B amplifier (Axon Instruments), filtered at 10 kHz and sampled at 20 kHz. The 10-mV liquid junction potential measured under the ionic conditions reported here was not corrected for.

Electrical stimulation was applied via a monopolar 2–3 KΩ Narylene-coated stainless steel microelectrode positioned on the rostrodorsal part of the STN. The anode was an Ag–AgCl pellet placed in the bath. Stimulation pulses consisted of 100–300 μA biphasic currents (200 μs cathodal followed by 200 μs anodal phase). The interval between consecutive pulses was 100 and 10 ms, over 30/20 s leading to stimulation frequencies of 10 (300 stimuli) and 100 Hz (2000 stimuli), respectively. In several experiments the following drugs were added to the ACSF: bicuculline (BCC) methiodide to block GABAa receptors (final concentration 50 μM), D(-)-2-amino-5-phosphonopentanoic acid (APV) (50 μM) and 6-cyano-7-nitroquinoxaline-2,3-dione (CNQX) (15 μM) to block NMDA and AMPA receptors, respectively.

### Data analysis

All off-line analyses were carried out using Matlab R2007b (Mathworks) and IgorPro 6.0 (WaveMetrics) on a personal computer. Data for each experiment were obtained from at least five rats. All results for each experiment were pooled and displayed as mean ± SD. The pre-stimulus average firing rate was calculated from spikes extracted from 30 s of continuous recording. Firing rate was defined as the time-dependent average firing rate aligned to the stimulation onset. Changes in firing pattern were determined through raster plots and peri-stimulus time histograms (PSTH) from each recording. A *t*-test gave the significance of changes before and after application of the drugs during stimulation (^*^p < 0.05 and ^**^p < 0.01).

## Results

**Figure 1:**
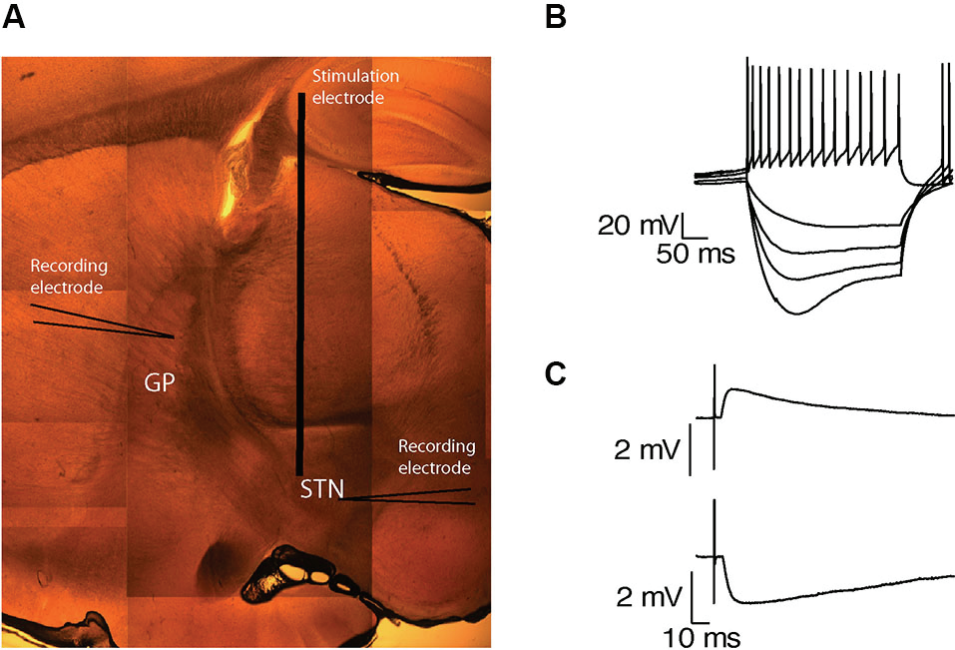
**Preservation of functional connectivity between the GP and the STN. (A)** A sagittal brain slice containing the STN and the GP. **(B)** Voltage responses of a subthalamic neuron to injection of positive and negative current steps. **(C)** EPSP recorded in the GP in response to STN stimulation (upper trace). IPSP recorded in the STN in response to GP stimulation (lower trace).

The STN was identified as a small oval nucleus above the internal capsule (Figure 1A). Neurons in this region were spontaneously active and were characterized by sag current induced by hyperpolarization (Figure 1B) and burst firing (*n* = 39), as previously reported (Nakanishi et al., 1987; Beurrier et al., 1999). The GP was identified rostral to the internal capsule and caudal to the striatum (Figure 1A). To assess the connectivity between both nuclei in the slices, subthalamic and pallidal neurons were labeled with biocytin, allowing us to track stained axons. Axons in both directions were best preserved when the sagittal slices were cut at an angle of 17° to the midline. To confirm the functional connectivity in each slice, we placed the stimulating electrode at the center of each nucleus and obtained whole-cell recordings from neurons in the other nucleus. As both subthalamic and pallidal neurons are spontaneously active, recorded neurons were hyperpolarized to identify synaptic activity. Single pulse stimulation of the STN evoked excitatory postsynaptic potentials (EPSPs) in the recorded GP neurons (Figure 1C). These EPSPs were abolished after application of APV and CNQX indicating a glutamatergic origin (data not shown). Single pulse stimulation of the GP evoked inhibitory postsynaptic potentials (IPSPs) in the subthalamic neurons, indicating the preservation of GABAergic axons from the GP to the STN (Figure 1C).

**Figure 2:**
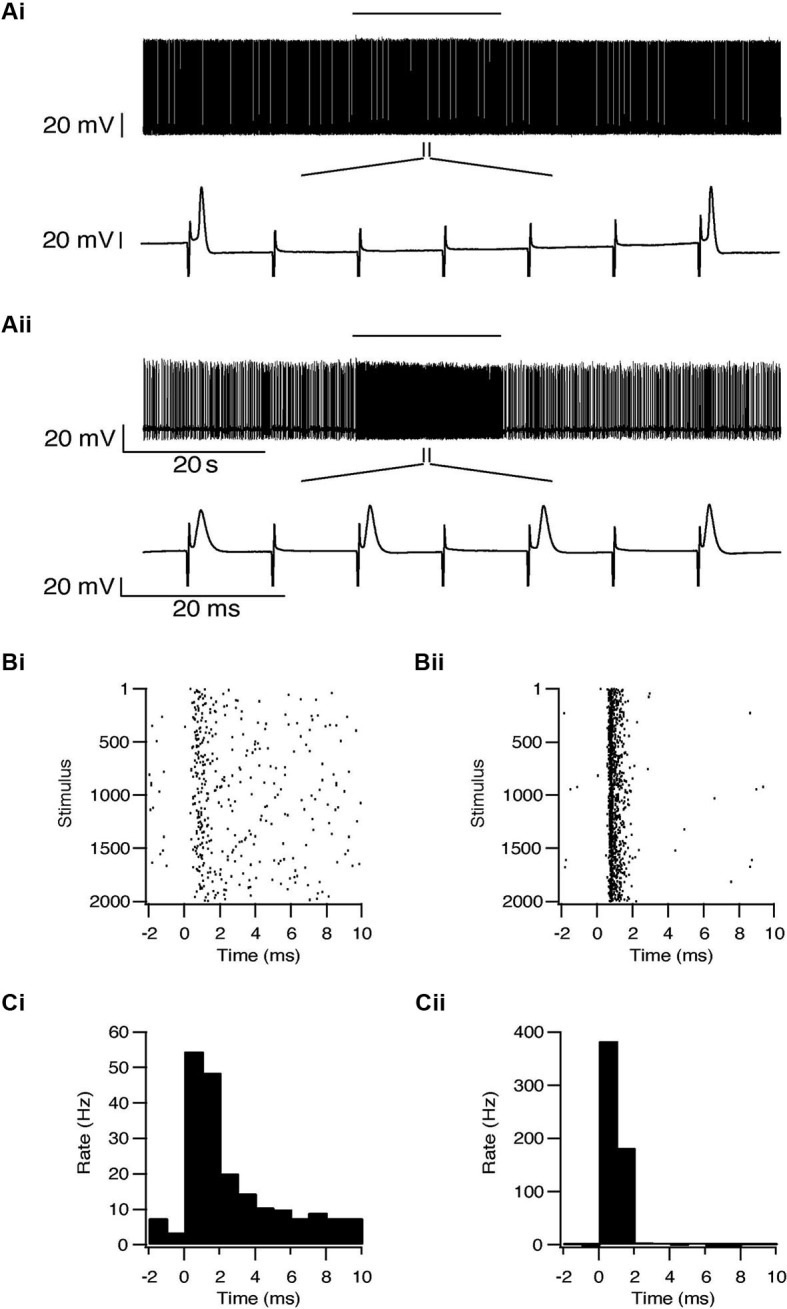
**Simultaneous recording from a subthalamic neuron (i) and a pallidal neuron (ii) during repetitive stimulation of the STN. (A)** Typical responses of a subthalamic and a pallidal neuron to 100 Hz stimulation of the STN. The stimulation artifact was removed to improve visualization of the neuronal activity. Horizontal lines indicate the stimulation period. **(B)** Raster plots of responses to each pulse constructed from all repetitions (*n* = 2000). **(C)** Peristimulus time histogram (PSTH).

**Figure 3:**
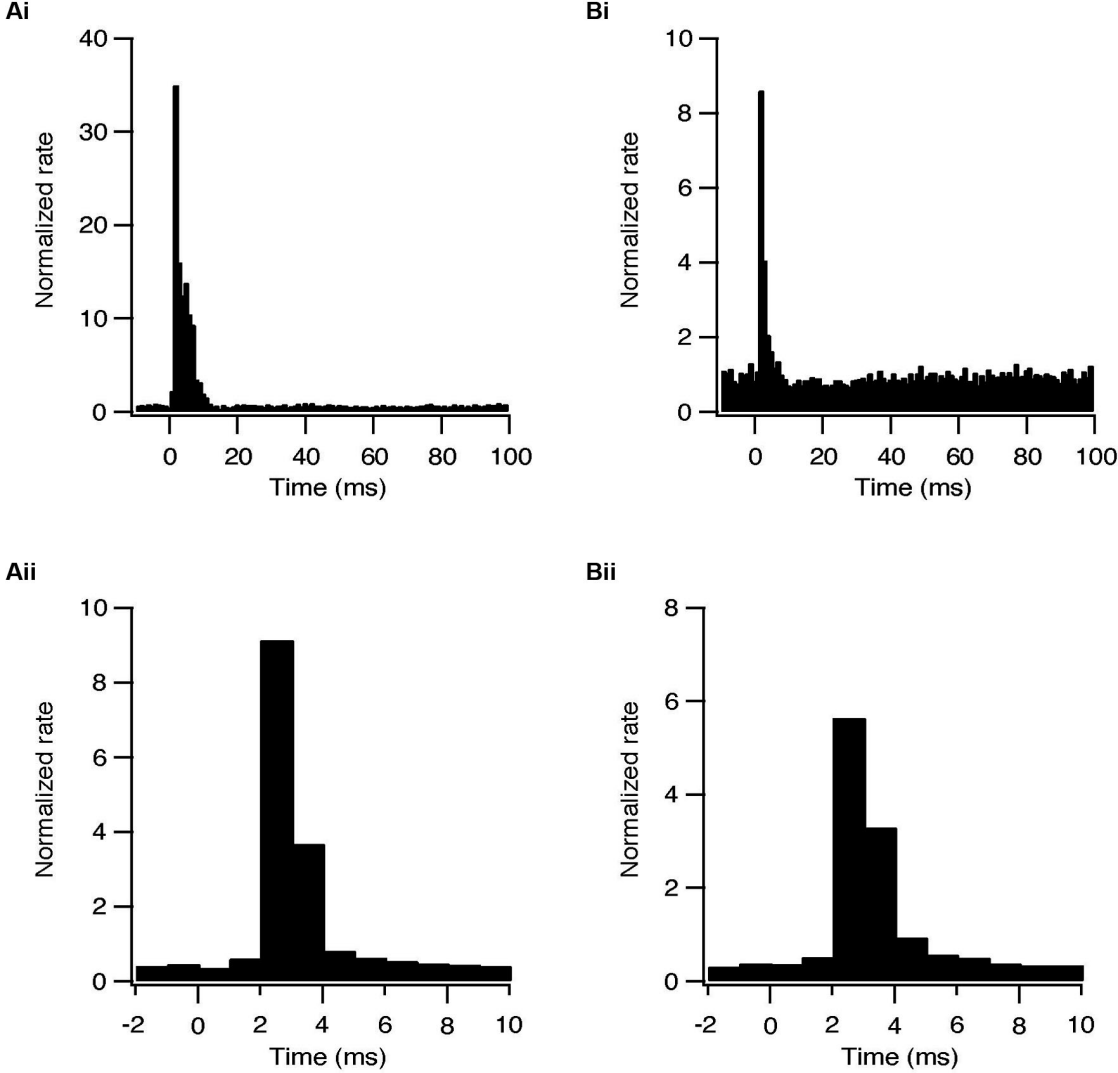
**Synaptically locked response during repetitive stimulation of the STN. (A)** Population PSTH of subthalamic neurons during 10 (i, *n* = 28) and 100 Hz (ii, *n* = 44) stimulation. **(B)** Population PSTH of pallidal neurons during 10 (i, *n* = 34) and 100 Hz (ii, *n* = 45) stimulation.

Whole-cell recordings were obtained from 47 subthalamic and 48 pallidal neurons during repetitive stimulation of the STN at different frequencies. All recorded neurons were labeled with biocytin showing that they lay within the boundaries of the appropriate nucleus. Figure 2A shows an example of such simultaneous recording of a subthalamic (i) and a pallidal neuron (ii) during 100 Hz stimulation of the STN. To investigate the effects of HFS, on the pallidal and subthalamic neurons we recorded simultaneously from both nuclei during 100 Hz stimulation. In order to identify changes in firing pattern, we calculated raster plots and PSTH from each recording (Figures 2B, C). HFS evoked significant changes in the firing pattern of most subthalamic (*n* = 44/47) and pallidal neurons (45/48) (Figure 3) as found *in vivo* (Hashimoto et al., 2003; Moran et al., 2011). During the stimulation the firing of neurons from both nuclei became partially locked to the stimulus pulses; population PSTHs showed that the STN and GP firing rate transiently increased at an average latency of 2.6 ± 1 ms and 2.5 ± 0.9 ms, respectively (Figure 3A). These latencies are consistent with *in vivo* findings (Kita and Kitai, 1991; Hashimoto et al., 2003; Moran et al., 2011). The effect of LFS was examined using stimulation at 10 Hz. Similar to the responses observed during HFS, most of the subthalamic (28/37) and pallidal (34/44) neurons showed a transient increase in firing rate at an average latency of 2.3 ± 0.8 ms and 2.5 ± 0.6 ms (Figure 3B).

**Figure 4:**
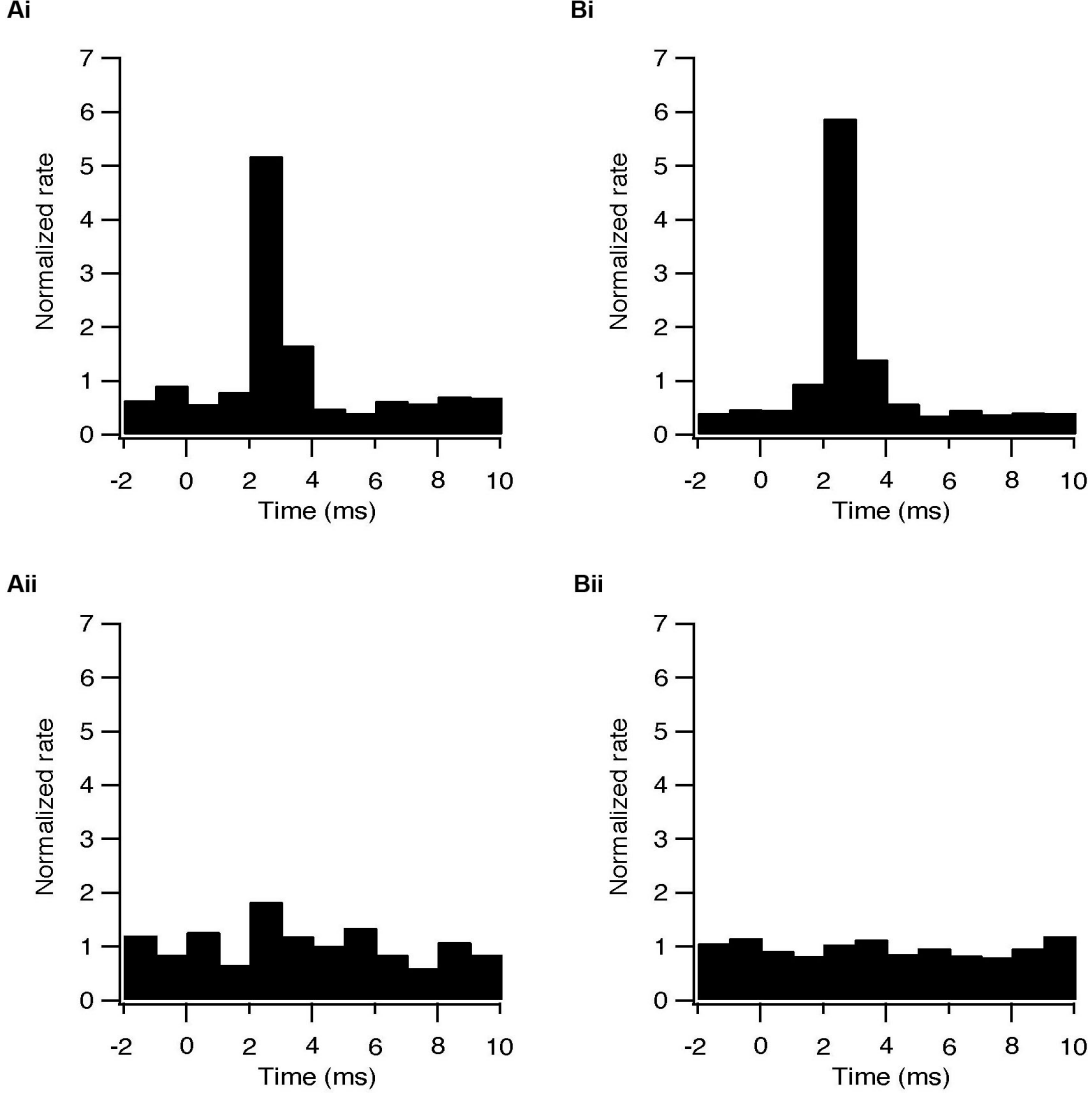
**Changes in firing pattern are of synaptic origin. (A)** Population PSTH of the response to each pulse of subthalamic (i, *n* = 6) and pallidal (ii, *n* = 6) neurons under control conditions. **(B)** Population PSTH of the same neurons following bath application of 50 μM APV and 15 μM CNQX, showing the absence of transient excitation in the STN (*p* < 0.05) and in the GP (*p* < 0.01) when glutamatergic conductance was blocked (i) sub thalamic and (ii) pallidal.

The early excitation in the STN evoked by the stimulation protocols could result from intrinsic activation of the subthalamic neurons or from the activation of afferent cortical and thalamic glutamatergic fibers. The early GP excitation could result from antidromic activation of pallidal axons or from the activation of subthalamic fibers. To determine the origin of this excitatory phase APV and CNQX were applied to the slices. These glutamatergic blockers completely abolished this excitatory response in both subthalamic and pallidal neurons (Figure 4). That is, rather than activating the neurons intrinsically, the stimulation results in activation of neurons in both nuclei via glutamatergic synapses.

**Figure 5:**
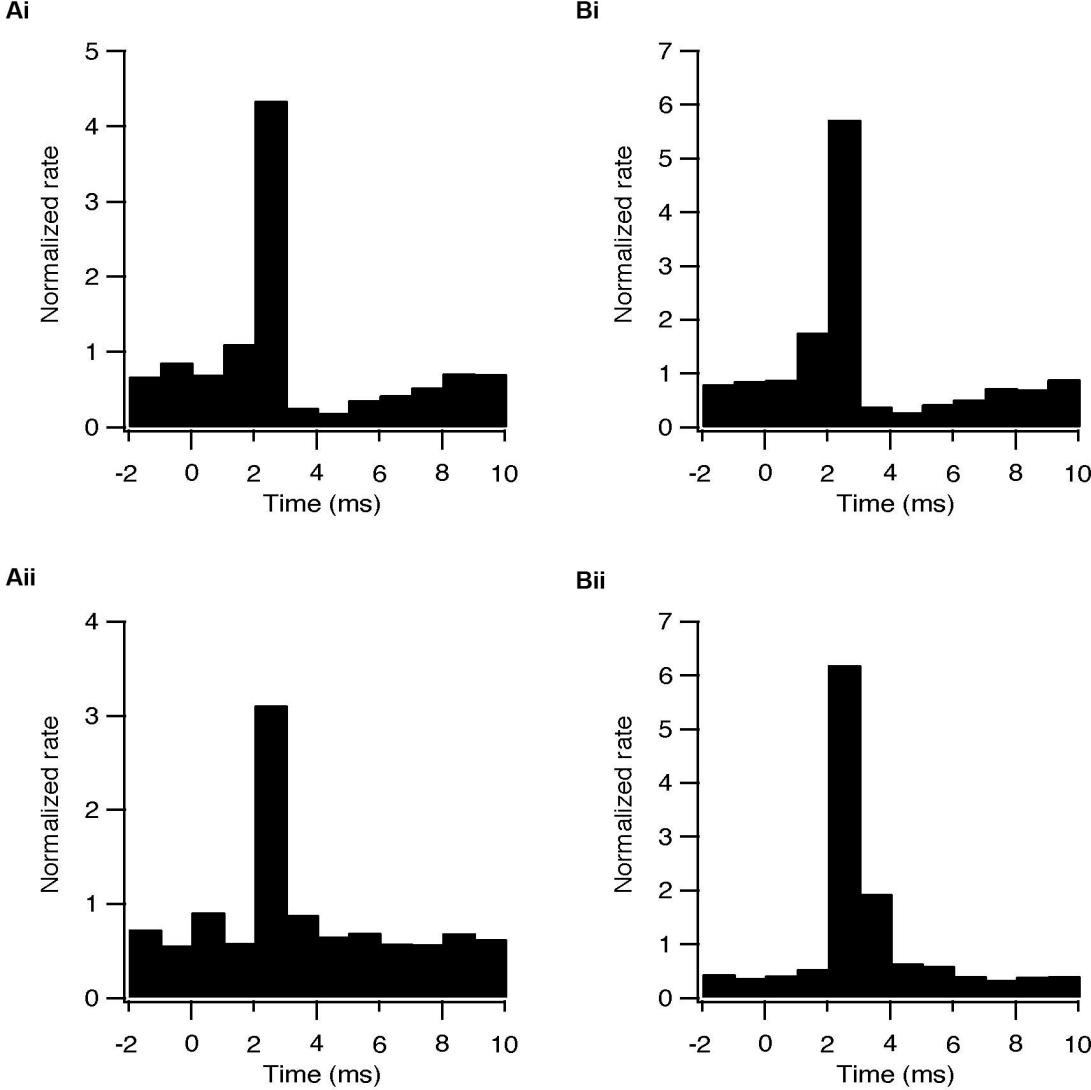
**Activation of GABAergic synapses during STN stimulation at 10 and 100 Hz. (A)** Population PSTH of the response to each pulse of subthalamic (i, *n* = 5) and pallidal (ii, *n* = 7) neurons under control conditions. **(B)** Population PSTH of the same neurons following bath application of 50 μM BCC, showing the absence of transient inhibition when GABAa receptors were blocked (*p* < 0.01), (i) sub thalamic and (ii) pallidal.

In some cases a transient inhibition followed the initial excitation evoked by each pulse. During HFS, the firing rate of 7/47 subthalamic (15%) and 9/48 pallidal neurons (19%) decreased to a minimum at an average latency of 3.7 ± 0.9 ms and 3.6 ± 0.5 ms, respectively (Figure 5A). Single pulses in LFS resulted in inhibition in 9 subthalamic (24%) and 19 pallidal (43%) neurons. This inhibitory phase was abolished by applying BCC (Figure 5B) and thus results from the delayed activation of GABAergic synapses. The lower prevalence of inhibition after HFS, compared with LFS, may be due to partial depression of GABAergic synapses due to the high frequency of stimulation.

**Figure 6:**
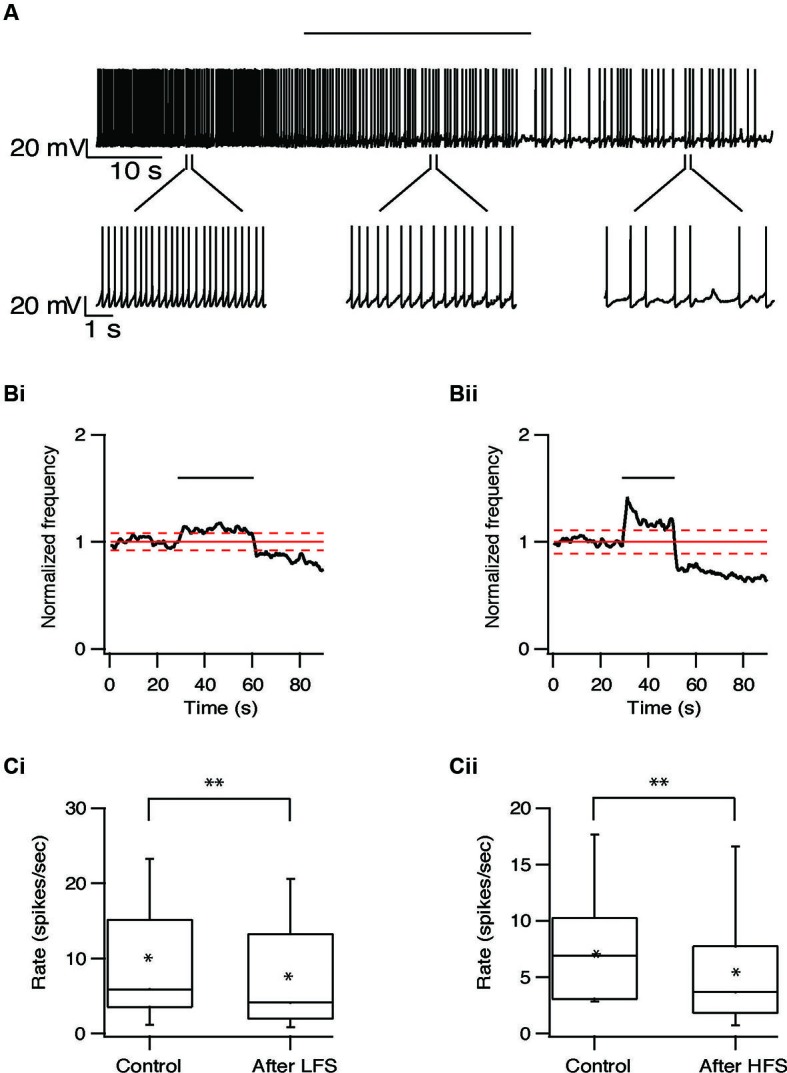
**Long-term depression of the STN induced by repetitive stimulation. (A)** Representative recording from a subthalamic neuron during 10 Hz stimulation of the STN. Stimulation artifact was removed to improve visualization of the neuronal activity. Horizontal line above indicates the stimulation period. **(B)** Changes in normalized population firing rate of subthalamic neurons in response to each pulse of 10 Hz (i, *n* = 22) and 100 Hz (ii, *n* = 28) stimulation of the STN. Thick and dashed red lines indicate the average ±2 SD of pre-stimulus frequency, respectively. **(C)** Box plots showing the differences in firing rate before and after 10 Hz (i) or 100 Hz (ii) stimulation (*p < 0.05 and **p < 0.01).

We next characterized the effect of repetitive STN stimulation on the firing frequency of the subthalamic and pallidal neurons. The population firing rate of the subthalamic neurons increased slightly during both HFS and LFS. In most subthalamic neurons, cessation of stimulation was followed by a prolonged decrease in firing rate. An example of such a prolonged decrease in the firing rate after LFS at 10 Hz is shown in Figure 6A. Following 10 Hz stimulation the firing rate of 22/37 subthalamic neurons was reduced by 31 ± 23% (*p* < 0.01); after 100 Hz stimulation the firing rate of 28/47 subthalamic neurons was reduced by 41 ± 26% (*p* < 0.01, Figures 6B, C). This prolonged inhibition was evident in cells from different areas within the STN and did not vary with the distance from the stimulation electrode.

**Figure 7:**
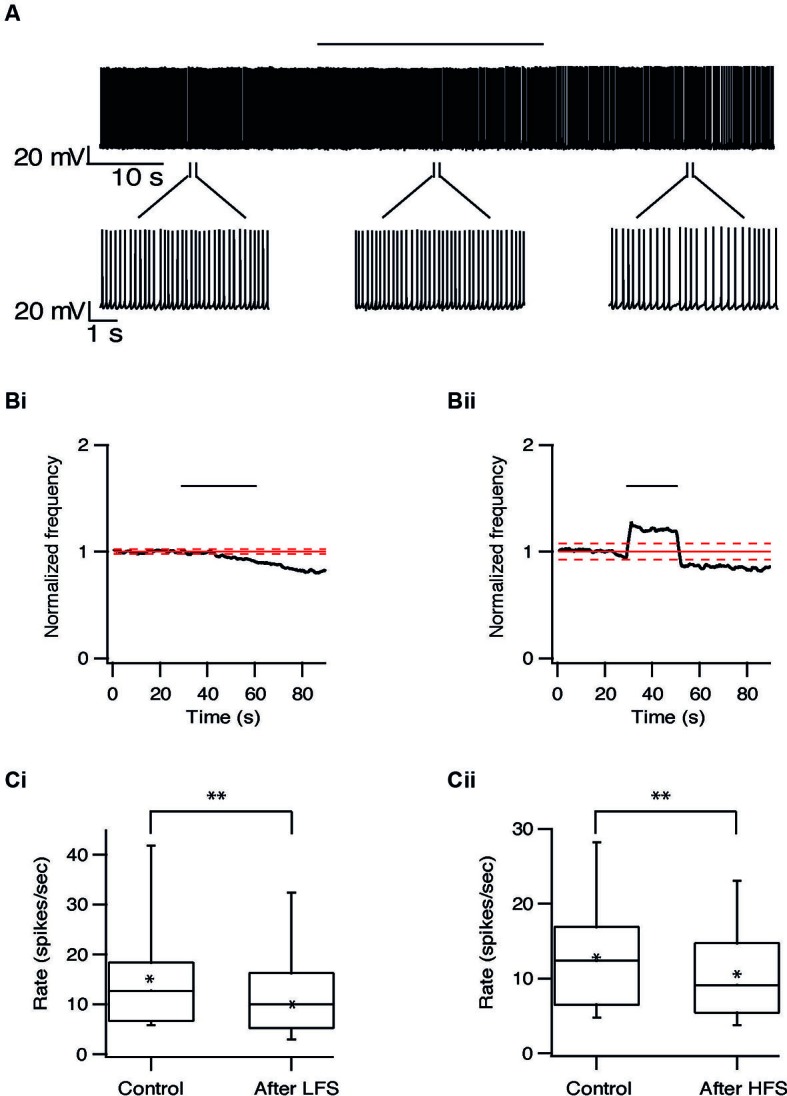
**Long-term depression in the GP induced by repetitive stimulation. (A)** Representative recording from a pallidal neuron during 10 Hz stimulation of the STN. Stimulation artifact was removed to improve visualization of the neuronal activity. Horizontal line above indicates period of stimulation. **(B)** Changes in normalized population firing rate of pallidal neurons in response to each pulse of 10 Hz (i, *n* = 20) and 100 Hz (ii, *n* = 25) stimulation of the STN. Thick and dashed red lines indicate the average ±2 SD of pre-stimulus frequency, respectively. **(C)** Box plots showing the differences in firing rate before and after 10 Hz (i) or 100 Hz (ii) stimulation (*p < 0.05 and **p < 0.01).

**Figure 8:**
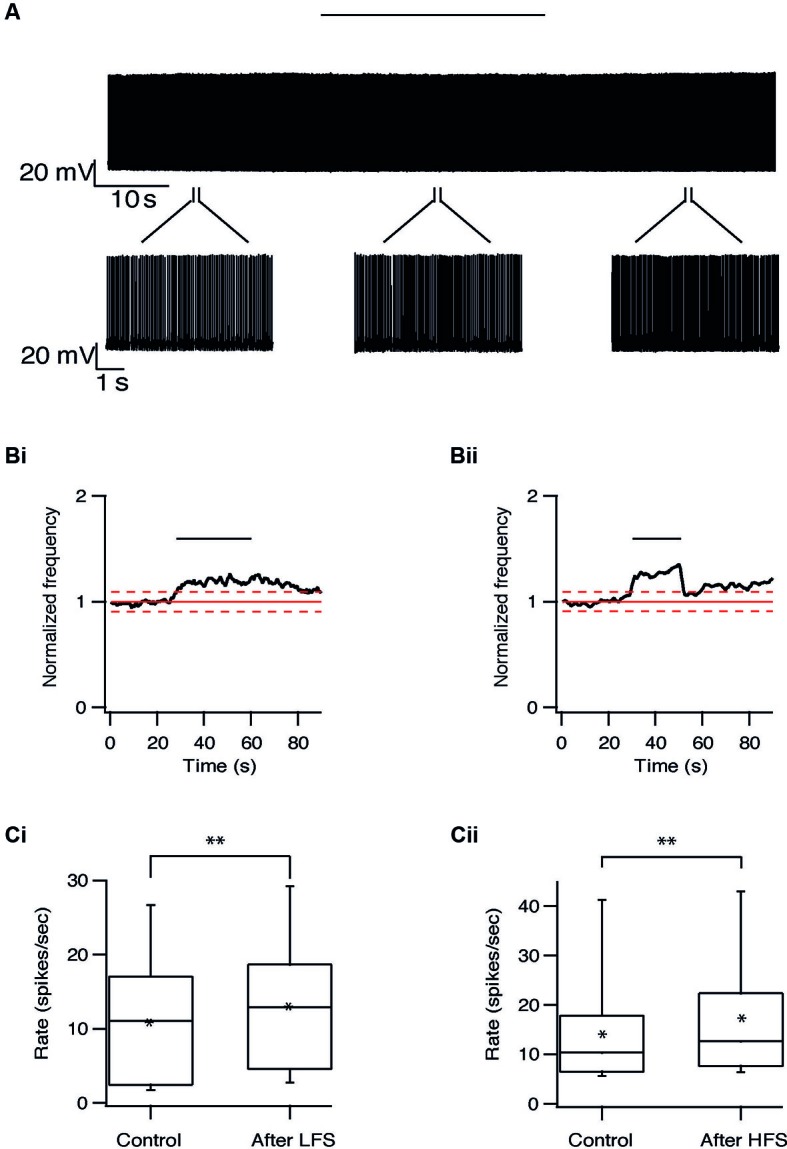
**Long-term excitation in the GP induced by repetitive stimulation. (A)** Representative recording from a pallidal neuron during 10 Hz stimulation of the STN. Stimulation artifact was removed to improve visualization of the neuronal activity. Horizontal line above indicates period of stimulation. **(B)** Changes in normalized population firing rate of pallidal neurons in response to each pulse of 10 Hz (i, *n* = 9) and 100 Hz (ii, *n* = 11) stimulation of the STN. Thick and dashed red lines indicate the average ±2 SD of pre-stimulus frequency, respectively. **(C)** Box plots showing the differences in firing rate before and after 10 Hz (i) or 100 Hz (ii) stimulation (*p < 0.05 and **p < 0.01).

Long-term effects were also observed in pallidal neurons. Similar to the responses recorded in the STN, the firing rate of 20/40 pallidal neurons was reduced by 22 ± 20% (*p* < 0.01) after 10 Hz stimulation and after 100 Hz stimulation the firing rate of 25/48 pallidal neurons was reduced by 21 ± 13% (*p* < 0.01) (Figure 7). As in the STN, there was no relation between the long-term response and the location of the pallidal neurons. However, in contrast to the STN, approximately a quarter of the pallidal neurons showed the opposite effect, a prolonged increase of firing rate which could be induced by both protocols (Figure 8). Following 10 Hz stimulation the firing rate of 9/40 pallidal neurons increased by 17 ± 11% (*p* < 0.01). Following 100 Hz stimulation the firing rate of 11/48 pallidal neurons increased by 24 ± 21% (*p* < 0.01).

## Discussion

This study characterized changes in firing of subthalamic and GP neurons during HFS and LFS of the STN. Both HFS and LFS led to significant short-term modulation of firing pattern in both nuclei (Figures 2, 3) through the activation of glutamatergic and GABAergic synapses (Figures 4, 5). LFS and HFS also induced similar long-term effects. In STN, both protocols induced long lasting suppression of firing rate in many neurons (Figure 6), while in the GP the same protocols could induce either long-lasting suppression or, less frequently, excitation (Figures 7, 8).

Both HFS and LFS activated glutamatergic and GABAergic synapses, thus reshaping the firing pattern of the subthalamic neurons (Figures 4, 5). None of the recorded cells was intrinsically activated by the stimulus pulses. These findings fit the changes in firing pattern found *in vivo* HFS studies (Hashimoto et al., 2003). It has also been shown *in vivo* that HFS of the STN raised the glutamate concentration in the GP. This suggests that HFS activates subthalamic neurons, which consequently excite pallidal neurons (Windels et al., 2000). We showed here that the excitation of both subthalamic and pallidal neurons was of glutamatergic origin. Previous findings showed that cortical afferents to the STN are activated antidromically during STN-HFS (Li et al., 2012). Our findings suggest that the activation of these glutamatergic fibers during STN-HFS or LFS consequently excite the subthalamic neurons.

During repetitive stimulation of the STN, the glutamatergic excitation after each pulse was followed by a GABAergic inhibition. Blocking the glutamatergic conduction blocked both excitatory and inhibitory phases (data not shown), indicating the dependence of the inhibitory GABAergic effect on activation of glutamategric fibers. It is possible that, due to differences in the basal activity of the glutamatergic and GABAergic afferents, the stimulus pulses directly activated only glutamatergic synapses, with the delayed GABAergic effect stemming from the increased activity of pallidal neurons. This may be due to differences in the basal activity of the glutamatergic and GABAergic afferents. The STN receives glutamatergic inputs from the cortex and thalamus and GABAergic inputs from the GP. Unlike the thalamic and cortical afferents, pallidal neurons fire spontaneously at relatively high rates and thus the GABAergic synapses are partially depressed (Atherton et al., 2013; Bugaysen et al., 2013). As a result, the stimulus pulses may only modulate the activity of the glutamatergic synapses, while the GABAergic synapses remain unaffected. This suggestion is supported by our finding that GABAergic inhibition was present in 24% of subthalamic neurons during 10 Hz stimulation, yet only in 15% neurons during 100 Hz stimulation.

We found no clear dynamic change in the neuronal activity during repetitive stimulation, however there was a significant depression of firing rate following either 100 or 10 Hz stimulation in most subthalamic and pallidal neurons (Figures 6, 7). In addition, both HFS and LFS induced prolonged increase of the firing rate in about a quarter of GP neurons but not in STN neurons (Figure 8). These results fit the different dynamic and static changes in firing rate induced by similar stimulation protocols applied to the GP (Erez et al., 2009; Bugaysen et al., 2011).

The differences in long-term responses between STN and GP (Figures 6–8) may arise from differences between the nuclei. Unlike the homogeneous neuronal population of the STN, the neurons in the GP can be classified into different populations by their electrophysiological and molecular characteristics (Cooper and Stanford, 2000; Bugaysen et al., 2010). These differences may account for the opposing long-term effects observed in the pallidal neurons. On the other hand, the differences in response may be explained by the inner connectivity of the GP. Pallidal neurons send collaterals to form inhibitory synapses within the GP (Sato et al., 2000; Sadek et al., 2007). We recently showed that a single pallidal neuron can modulate the postsynaptic firing rate of other neurons in the GP (Bugaysen et al., 2013). It is thus possible that the different long-term responses were recorded from neurons receiving different synaptic inputs. One may expect that pallidal neurons receiving direct input from the STN exhibit a prolonged depression similar to that in the subthalamic cells. As the activity of these neurons decreases, other pallidal neurons may increase their firing rate due to disinhibition. This hypothesis remains to be investigated.

As the long-lasting effects observed here after repetitive stimulation were independent of frequency, they may be due to biochemical processes rather than inactivation of the stimulated neurons. Shen et al. (2003) reported that that HFS of the STN *in vitro* induced either long-term depression or long-term potentiation in the subthalamic synapses, implicating synaptic plasticity mechanisms. Here we found no excitatory effect in the STN. It should be pointed out that the experiments reported here were performed in slices obtained from normal rats, and may not represent the effects of STN-HFS and LFS in dopamine depleted preparations. As long-term plasticity mechanisms may be dopamine dependent (Yamawaki et al., 2012; Dupuis et al., 2013), similar measurements from dopamine-depleted slices are further required.

HFS is commonly thought to alleviate PD symptoms through inhibiting STN neurons. *In vitro* studies have shown that HFS of the STN caused complete cessation of firing during or following stimulation (Beurrier et al., 2001). An *in vivo* study showed decreased firing rate of subthalamic neurons during, but not after, HFS of the STN in 6-hydroxy dopamine (6-OHDA) lesioned rats (Filali et al., 2004). These studies indicate HFS has an inhibitory effect on the STN and suggested that this may plays a role in alleviating PD symptoms. This hypothesis is contended by our results, which, as far we know, are the first demonstrating similar inhibitory effects induced by LFS of the STN. LFS shows no therapeutic effect in PD and may even worsen the symptoms. Therefore the mechanisms underlying the therapeutic effect of HFS in the STN remain to be resolved.

## Conflict of interest statement

The authors declare that the research was conducted in the absence of any commercial or financial relationships that could be construed as a potential conflict of interest.
